# Splay states in networks of identical integrate-and-fire neurons

**DOI:** 10.1186/1471-2202-15-S1-P91

**Published:** 2014-07-21

**Authors:** Jan R Engelbrecht, Bolun Chen, Renato Mirollo

**Affiliations:** 1 Physics Department, Boston College, Chestnut Hill, MA 02467, USA; 2 Mathematics Department, Boston College, Chestnut Hill, MA 02467, USA

## 

We develop an analytic framework to investigate the stability of splay states in infinite networks of identical integrate-and-fire neurons coupled through synaptic pulses. More specifically we perform a linear stability analysis of the splay state probability distribution whose dynamics is governed by an appropriate Fokker Planck equation. For exponentially decaying synaptic pulses the splay state is unstable for excitation and stable for inhibition. For excitatory alpha-function pulses the splay state becomes stable for sufficiently large decay times and we find an analytic expression for the boundary of stability. This large decay time stability is analogous to the stability of synchronous states for inhibition studied by van Vreeswijk, Abbott and Ermentrout [1]. For inhibitory alpha-function pulses the splay state is unstable, but for smaller decay times (when there is no stable synchronous state) the splay state exhibits a remarkable attracting meta-stable transient. We complement our analytic framework with numerical simulations on finite networks.
